# Cardiorespiratory Fitness and Peak Torque Differences between Vegetarian and Omnivore Endurance Athletes: A Cross-Sectional Study

**DOI:** 10.3390/nu8110726

**Published:** 2016-11-15

**Authors:** Heidi M. Lynch, Christopher M. Wharton, Carol S. Johnston

**Affiliations:** Arizona State University, School of Nutrition and Health Promotion, Phoenix, AZ 85004, USA; Christopher.Wharton@asu.edu (C.M.W.); Carol.Johnston@asu.edu (C.S.J.)

**Keywords:** vegetarian, endurance, VO2 max, dynamometer, protein, sustainability, torque, body composition, Dual X-ray Absorptiometry (DXA)

## Abstract

In spite of well-documented health benefits of vegetarian diets, less is known regarding the effects of these diets on athletic performance. In this cross-sectional study, we compared elite vegetarian and omnivore adult endurance athletes for maximal oxygen uptake (VO2 max) and strength. Twenty-seven vegetarian (VEG) and 43 omnivore (OMN) athletes were evaluated using VO2 max testing on the treadmill, and strength assessment using a dynamometer to determine peak torque for leg extensions. Dietary data were assessed using detailed seven-day food logs. Although total protein intake was lower among vegetarians in comparison to omnivores, protein intake as a function of body mass did not differ by group (1.2 ± 0.3 and 1.4 ± 0.5 g/kg body mass for VEG and OMN respectively, *p* = 0.220). VO2 max differed for females by diet group (53.0 ± 6.9 and 47.1 ± 8.6 mL/kg/min for VEG and OMN respectively, *p* < 0.05) but not for males (62.6 ± 15.4 and 55.7 ± 8.4 mL/kg/min respectively). Peak torque did not differ significantly between diet groups. Results from this study indicate that vegetarian endurance athletes’ cardiorespiratory fitness was greater than that for their omnivorous counterparts, but that peak torque did not differ between diet groups. These data suggest that vegetarian diets do not compromise performance outcomes and may facilitate aerobic capacity in athletes.

## 1. Introduction

Vegetarian diets are increasingly being adopted for a variety of reasons including health, sustainability, and ethics-related concerns. Adherence to a vegetarian diet has been associated with a reduced risk of developing coronary heart disease [1], breast cancer [2], colorectal cancers [3], prostate cancer [4], type 2 diabetes [5], insulin resistance [6], hypertension [7], cataracts [8] and dementia [9]. Vegetarians also typically have a lower body mass index (BMI) [10] and an improved lipid profile [11]. In addition to promoting physical health, reducing or eliminating meat from the diet is environmentally advantageous since producing meat requires more land, water, and energy resources than growing plants for food [12], and producing meat creates more greenhouse gases compared to a plant-based diet [13,14].

In spite of the many health aspects of vegetarian diets some concern has been raised pertaining to the nutrient adequacy of vegetarian diets for supporting athletic performance. Vegetarian diets are typically lower in vitamin B12, protein, creatine, and carnitine [15,16], and iron and zinc from plant sources are less bioavailable than from meat sources [17]. However, vegetarian diets are typically higher in carbohydrate and antioxidants [18,19], which may be advantageous for athletic performance, particularly for endurance activities [20].

Despite these issues, little research directly examining vegetarian diets and athletic performance is available. There have been mixed results regarding hypertrophic potential when comparing vegetarian diets with omnivore diets during resistive exercise training; however, in all cases these differences did not translate to differential strength gains at the completion of the trials [21,22,23,24]. Adoption of a lacto-ovo vegetarian (LOV) diet for six weeks did not significantly affect endurance performance among a group of trained, male endurance athletes, in spite of a decrease in total testosterone while on the vegetarian diet [25]. There were also no group differences between 20 participants adopting an LOV diet compared to maintaining their usual omnivorous diet in terms of muscle buffering capacity in conjunction with sprint training for five weeks [26]. These studies provide some insight into the effect of a vegetarian diet on athletic performance. However, a considerable limitation in many of these studies is the inclusion of participants who typically consume meat but subsequently adopt a vegetarian diet only for the duration of the study rather than comparing participants who have adhered to a vegetarian or meat-containing diet long-term.

In a 1986 observational trial, Hanne and colleagues compared athletes who had maintained either an LOV or omnivore diet for at least two years and found no group differences for aerobic or anaerobic capacity [27]. However, aerobic capacity was estimated using cycle ergometry and predicted VO2 max, and strength or torque were not measured. Moreover, body adiposity was estimated using skinfold thickness. Given the current interest in vegetarian diets, in terms of both long-term health and environmental benefits, it is important to reaffirm, using leading-edge technology, that high-level athletic performance is supported by these diets.

The purpose of the present cross-sectional study was to examine body composition and performance measures in vegetarian and omnivore adult endurance athletes who had adhered to their respective diet plans for at least three months. Body composition, including visceral adiposity, was measured using dual-energy X-ray absorptiometry (DXA), leg strength was measured using a dynamometer, and aerobic capacity was determined using the Bruce protocol treadmill test. It was hypothesized that there would be no differences between groups on any parameters.

## 2. Materials and Methods

### 2.1. Participant Recruitment 

Healthy men and women, both vegetarians and omnivores, were recruited through advertisements on Stevebay.org (a popular website for endurance athletes), Facebook, and through word of mouth. Participants were either on a competitive club sports team at a National Collegiate Athletic Association (NCAA) Division 1 university or training for a major endurance race (such as a marathon, triathlon, cycling race, or other ultra-endurance event). An equal number of omnivore and vegetarian athletes were enrolled in the study between the ages of 21–58 years (35 per group); however, answers to diet questions indicated that eight of the vegetarians ate meat on occasion, and these subjects were reclassified as omnivores. Participants completed a health history questionnaire and were excluded if they had any chronic disease. All participants had the study verbally explained to them and provided their written consent; this study was approved by the Institutional Review Board at Arizona State University, number HS1211008557. Study recruitment and all study measurements took place between August and November 2015.

### 2.2. Experimental Approach

In this cross-sectional investigation participants completed all study measurements in a single visit. Prior to the visit, participants completed a seven-day food log. Fifty-seven out of seventy participants returned completed food logs, all of which were used in dietary analysis using Food Processor SQL Nutrition and Fitness Software by ESHA Research, Inc. (version 10.11.0, Salem, OR, USA). Height and body mass were measured using a SECA directprint 284 digital measuring station when participants were wearing light clothing and no shoes. Participants also completed a full-body DXA scan (Lunar iDXA, General Electric Company, East Cleavland, OH, USA), which was conducted by a certified radiology technologist.

Maximal oxygen uptake was determined by following the Bruce protocol [28] on a Trackmaster TMX425C treadmill using the Parvo Medics TrueOne 2400 (Sandy, UT, USA) metabolic measurement system. Prior to beginning the test, participants were instructed how to report their fatigue level using the Borg rating of perceived exertion (RPE) scale [29]. When asked by a research assistant, they reported their RPE at the end of each minute of the test by pointing to a printed Borg RPE chart being held by a research assistant. Participants were verbally encouraged by the research team to push as long as they could and to try to reach a true maximal effort. Handrail support was not allowed during the test. Maximal respiratory exchange ratio (RER) was recorded to help determine whether subjects had reached a “true” maximal effort during the test. Maximal RER values of ≥1.1 were considered indicative of true maximal oxygen uptake [30,31]. Peak oxygen uptake reported is the highest oxygen uptake measured during the test. 

Finally, participants completed a series of leg extensions and flexions on the HumacNorm isokinetic dynamometer (Computer Sports Medicine Inc. (CSMi, Stoughton, MA, USA) at 60 degrees per second (d/s), 180 d/s, and 240 d/s. Participants were familiarized with the protocol and conducted one practice repetition at each speed prior to performing three maximal effort repetitions at each speed. All sets, including practice repetitions, were performed on both legs, and self-reported dominant side was recorded. Participants moved from the VO2 max test immediately into the dynamometer testing, and there were 30 s of rest between sets on the dynamometer.

### 2.3. Statistical Analyses

Based on the data of Hanne et al. [27], at 80% power and an alpha level of 5%, 15 participants per group would be needed to detect a 10% difference in strength and 80 participants per group would be needed to detect a 10% change in aerobic capacity between groups. Data were analyzed for normality and log transformed if necessary, and outliers (values > 3 standard deviations (SD) from the mean) were removed prior to data analyses. Data reported are the mean ± SD, and participant characteristics are displayed by gender and diet group. A 2-way analysis of variance (ANOVA) analysis was used to determine differences between diet groups for participant characteristics followed by an independent *t*-test for post-hoc examination by diet within gender if indicated. Dietary data are reported by group, and a general linear model analysis was used to examine differences between groups controlling for gender. Data were analyzed using the Statistical Package for Social Sciences (SPSS) 23.0 for Mac (SPSS, Inc., Chicago, IL, USA).

## 3. Results

In the vegetarian group, 24 of the 27 participants (89%) had adhered to a vegetarian diet for >2 years. Of the remaining three participants, the diet had been followed for three, six, or eleven months. Fifteen of the vegetarians were vegans (nine men and six women), and twelve were lacto-ovo vegetarians (five men and seven women).

There were no significant age or gender differences between groups (Table 1). Significant differences were noted between diet groups for body mass and for lean body mass (LBM): female vegetarians tended to have a lower total body mass and LBM compared to the female omnivores (−11% and −7% respectively). Adiposity, however, did not differ between diet groups. Physical activity levels, recorded as kcal·kg^−1^·week^−1^, were 20% higher for vegetarians compared to omnivores (*p* = 0.018) (Table 1). Maximal oxygen uptake (mL/kg/min) differed significantly between diet groups, and post-hoc analyses revealed a significantly greater aerobic capacity in the female vegetarians in comparison to the female omnivores (+13%, *p* < 0.05) (Table 1); however, absolute maximal oxygen uptake (L/min) did not differ between diet groups. Peak torque when doing leg extensions was not different between diet groups. The 7-day diet records revealed several differences in nutrient intake between diet groups. Although total energy intakes were similar between the diet groups, the vegetarians consumed more carbohydrate, fiber, and iron daily compared to omnivores (Table 2). However, daily intakes for protein, saturated fat, cholesterol, vitamin B12, and selenium were lower among the vegetarians in comparison to the omnivores.

## 4. Discussion

Results from this study indicate that compared to their omnivore counterparts, vegetarian endurance athletes have comparable strength as indicated by leg extension peak torque, and possibly a greater degree of aerobic capacity, particularly in females, as indicted by a progressive maximal treadmill test to exhaustion. Dietary intake on several key nutrients differed considerably between groups. Some, but not all, results are consistent with previous reports.

Our study is significant for its increased rigor in measurement assessments compared to previous comparisons of vegetarian and omnivore athletes. We determined maximal oxygen uptake by a graded test to exhaustion on a treadmill instead of predicting VO2 max using a cycle ergometer, as recommended by Shepard and colleagues [32]. Additionally, we measured body composition using a DXA scan, currently regarded as the clinical gold standard for body composition assessment, instead of skinfolds [33]. Finally, we assessed both athletic performance and nutrient intake differences between vegetarians and omnivores, whereas most previously published studies focus exclusively on one of these areas.

### 4.1. Body Mass and BMI

Like other studies of vegetarians in the general population, vegetarian participants in the present study had significantly lower body mass compared to omnivores [10,34]. This is in spite of the fact that our study included participants engaged in considerable endurance activities, which could be very different in multiple ways from the general population. One prior study in athletes, conducted by Hanne et al. compared vegetarians and omnivores anthropometrically and found no significant differences between groups for weight [27]. It is noteworthy that the athletes in the Hanne et al. study included football, basketball, and water polo players in addition to endurance athletes. 

### 4.2. Lean Body Mass

LBM was significantly lower for the vegetarian athletes compared to their omnivore counterparts, a difference which was most prominent among the female participants with female vegetarian athletes possessing 7% less LBM as compared to the female omnivore athletes. In spite of this, there were no significant differences in body fat percentage or BMI between groups. To our knowledge, this is the first study to examine lean body mass differences between vegetarian and omnivore athletes. It is important to note, however, that this difference in lean body mass did not translate into differential peak torque on the leg extension.

Although other studies have not assessed lean body mass of vegetarian athletes specifically, Campbell and colleagues compared resistance-training induced changes in lean body mass and strength between groups assigned to either an omnivorous diet or a lacto-ovo-vegetarian diet for the duration of the study and found that, in spite of differential lean body mass gains, the two groups increased strength similarly [21]. Conversely, a 12-week training study by Haub and colleagues showed no significant differences in strength, body composition, or muscle cross-sectional area between groups assigned to either a lacto-ovo-vegetarian or beef-containing diet. 

### 4.3. Body Fat Percent and Visceral Adipose Tissue (VAT)

Contrary to the female vegetarian athletes in Hanne’s group, no significant differences in body fat percentage were found between vegetarian and omnivore athletes in this study. Additionally, there were no significant differences between groups for visceral adipose tissue (VAT). Participants in the present study had VAT values above those reported for similar aged healthy lean sedentary adults (~250 cm^3^), both omnivores and vegetarians [35,36], but lower than those noted for older adults (1000–1560 cm^3^) [37]. Although there are no standard reference ranges for VAT, values near 1000 cm^3^ were associated with BMI values near 25 kg/m^2^ and values > 300 cm^3^ have been suggested as predictive of risk for metabolic syndrome in young adults [36,37]. As technology permitting quantification of visceral adipose tissue is relatively new for research purposes, this study contributes to the emerging literature by providing VAT values for athletes. VAT and BMI is strongly correlated in this study (*p* = 0.742), a factor that may be important for estimating VAT inexpensively without a DXA scan.

### 4.4. VO2 Max

Unlike athletes in Hanne’s study, vegetarians in the present study had significantly higher maximal oxygen uptake than their omnivore counterparts [27]. This difference was most predominant in the female participants with a 13% greater VO2 max score for the female vegetarians as compared to the female omnivores, but this difference was not observed for absolute VO2 max (L/min), which suggests that body weight factored into this difference. This gender difference is intriguing and merits further investigation in future studies. One potential reason that athletes in the present study had higher VO2 max values than those in Hanne’s study may be due to the difference between cycle ergometry and treadmill testing methods. However, it is possible that the athletes in our study simply were more trained and that diet effects on differences in VO2 potential emerge only at higher levels of fitness.

Other work that contributes to our understanding of aerobic and anaerobic performance differences by diet include the study of Hietavala et al. that found no significant difference in time to exhaustion (albeit a higher oxygen uptake at a given percent of maximal oxygen consumption) between participants following a low-protein vegetarian diet compared to a mixed diet [38]. Subjects in this study adhered to the low protein vegetarian diet (0.80 ± 0.11 g of protein per kilogram of body mass (g/kg) vs. 1.59 ± 0.28 g/kg on their normal diet) for four days before being tested on a cycle ergometer. As this study did not use participants who practiced vegetarianism outside of the study, and the amount of protein that subjects were allowed to consume on the vegetarian diet was restricted, true differences between vegetarians and omnivores may not be evident. Baguet et al. found no differences in repeated sprint ability between participants following a vegetarian or mixed diet for five weeks; again, these subjects were not following a vegetarian diet long-term [26]. Raben et al. found no differences in maximal oxygen uptake among subjects after adoption of a lacto-ovo vegetarian diet for six weeks [25]. However, the major disadvantage of interpreting results of these studies for vegetarian athletes is that participants in these studies only adhered to a vegetarian diet briefly for the duration of the study.

### 4.5. Peak Torque

Similar to the Hanne et al. study that compared the power output of vegetarian and omnivore athletes [27], we found no significant differences by diet in terms of peak torque using leg extensions. Other studies in untrained older men that have examined strength development over time in response to a training program have found mixed results when comparing participants following a vegetarian or mixed diet [21,24]. This is noteworthy, particularly since strength and lean body mass were strongly correlated (*r* = 0.764) in the present study, as well as the fact that omnivores had significantly more lean body mass vs. the vegetarians. A nonsignificant trend for omnivores to produce higher peak torque is observed, however. It is conceivable that the omnivore diet pattern may be preferred for sports that rely on greater lean mass, and subsequently peak torque. To further investigate this, future work ought to examine if strength can be increased similarly by vegetarian and omnivore athletes engaged in strength training (not just by participants following a vegetarian diet for a few weeks).

### 4.6. Nutrient Intake

Nutrient intake was calculated from food and beverage intakes only and did not include any supplements. There were no significant differences in caloric intake or total fat intake between vegetarians and omnivores. However, vegetarians reported significantly more dietary carbohydrate (both in terms of absolute intake and as a percent of daily calories), fiber, and iron intake. Omnivores consumed more dietary protein (both in terms of absolute intake and as a percent of daily calories), saturated fat, cholesterol, and vitamin B12. However, when expressed relative to body mass, there were no differences in dietary protein intake.

That vegetarians and omnivores in the present study did not differ in terms of caloric intake is consistent with findings by Janelle and Barr from their comparison of 45 vegetarian and omnivore women [16], yet it is in contrast to results from Calkins and colleagues who compared 50 vegetarian, vegan, and omnivores. They found vegetarians consumed about 200 fewer kcal than omnivores [19]. These studies were both in the general population, not specifically with athletes. Calkins et al. also reported that omnivores consumed more fat than vegetarians, a fact that partially contributed to the higher caloric intake. This too is in contrast to the findings in the present study which found no significant difference either in grams of fat consumed or the percent contribution of fat to the daily calorie intake, even though saturated fat was significantly higher in omnivorous diets. Other studies involving the general population have also reported omnivores eating more energy and total fat than vegetarians [10,39,40,41].

Higher carbohydrate (when expressed either as an absolute amount or as a percent of total daily calories) and fiber intake among vegetarians in comparison to omnivores in the present study is consistent with findings in other studies [10,39,41,42,43,44]. As these studies have been conducted in the general population, the present study contributes to the literature by demonstrating that this dietary pattern can be extended to endurance athletes as well. One study by Janelle and Barr stands in contrast to these findings, as they did not find significant differences in carbohydrate or fiber intake between vegetarian and omnivore women; those participants were not athletes [16]. That vegetarians in the present study consumed more carbohydrates than omnivores is notable since they are all athletes, and the importance of carbohydrates for exercise is well-established [45,46,47].

Like the present study, other studies have also reported that vegetarians consume less protein (both absolute intake and as a percent of the daily calories) [10,16,39,42] and vitamin B12 [40,48] than omnivores. Our study contributes to the literature since other reports have been in the general population instead of within athletic groups. Of note, though, differences in dietary protein intake are not significant when expressed relative to body mass, which is typically the preferred method for recommending protein for athletes [47]. Nonetheless, dietary protein intake was weakly correlated with peak torque (*r* = 0.359, *p* = 0.006) in the present study, and dietary protein intake was moderately correlated with lean body mass (*r* = 0.415, *p* = 0.001). Expectantly, lean body mass was strongly correlated with peak torque (*r* = 0.764, *p* < 0.001). Hence, it is conceivable that protein intake could influence strength if intakes had been inadequate. In the present evaluation, protein intakes in the vegetarian participants averaged 1.2 g/kg body mass, which falls in the recommended range for athletes [47,49].

There are conflicting findings in the rest of the literature regarding whether omnivores or vegetarians consume more iron. The Wilson et al. study of vegetarian men found that vegetarians consumed more iron [41], but Ball and Bartlett reported no difference in dietary iron intake between female vegetarian and omnivores [50]. Clary et al. compared 1475 vegans, vegetarians, semi-vegetarians, pescetarians, and omnivores and also showed that vegetarians consume more iron than omnivores [39]. Although vegetarians consumed more iron than omnivores in the present study, iron bioavailability was likely reduced as has been shown in other trials [17]. Dietary intakes of zinc did not vary by diet group herein, but generally the literature suggests that vegetarians consume somewhat less dietary zinc than omnivores [16,51,52,53]. The lower intakes of selenium by vegetarians in comparison to omnivores has also been reported by others and reflects the low levels of selenium in plant foods relative to flesh foods [54,55].

### 4.7. Limitations

In addition to the small sample size, limitations to the study include the variable level of experience of the athletes for their respective sports, and related fitness levels. Although most participants were training for and competing in races such as marathons, Ironman-distance triathlons, and competitive cycling, there were a few participants who were training for shorter distance races. However, this variation makes results more generalizable to athletes of various fitness levels. 

### 4.8. Future Directions

Future work is needed to compare vegetarian and omnivore endurance athletes’ performance on events more similar to actual sporting events (such as time trials or peak power on a cycle ergometer) and probe differences by type of vegetarian diet (lacto-ovo vegetarian or vegan). Additional work is needed to explore the adequacy of long-term adherence to vegetarian and vegan diets for supporting development of lean body mass.

## 5. Conclusions

Our cross-sectional comparison of vegetarian and omnivore adult endurance athletes shows higher maximal oxygen uptake values among vegetarians and comparable strength, in spite of anthropometric and dietary differences. This study suggests that following a vegetarian diet may adequately support strength and cardiorespiratory fitness development, and may even be advantageous for supporting cardiorespiratory fitness. Certainly many factors affect an athlete’s sports performance, and there is no dietary substitute for quality training. However, our study contributes to the literature about cardiorespiratory and strength comparisons between vegetarian and omnivore endurance athletes, and may provide a rationale about the adequacy of vegetarian diets for sport performance. As this was a small cross-sectional study using endurance athletes, larger intervention trials are necessary to bolster conclusions about adequacy of vegetarian diets to support performance in strength and power-focused sports.

## Figures and Tables

**Table 1:** Participant characteristics by diet group (vegetarian, VEG; omnivorous, OMN) ^1^.

	VEG			OMN	*p*
Measure	Male (14)	Female (13)	Male (26)	Female (17)	
Age, year	36.1 ± 10.2	36.7 ± 7.7	38.0 ± 10.0	37.1 ± 8.7	0.608
Body mass, kg	73.3 ± 14.8	58.3 ± 7.6 **	78.0 ± 11.0	65.4 ± 11.6	**0.043**
BMI, kg/m^2^	24.0 ± 4.4	21.8 ± 2.5	24.8 ± 2.6	23.5 ± 3.8	0.123
Lean mass, kg	56.3 ± 7.4	42.0 ± 4.9 **	60.2 ± 7.3	45.4 ± 5.1	**0.026**
Waist, cm	81.6 ± 10.7	69.0 ± 14.8	85.2 ± 7.4	73.8 ± 8.2	0.093
Body fat, %	19.2 ± 6.5	25.5 ± 4.2	19.2 ± 6.4	26.9 ± 8.1	0.659
Visceral fat, cm^3^	447.4 ± 419.8	110.4 ± 123.0	538.5 ± 404.3	206.4 ± 254.6	0.656
METS, kcal·kg^−1^·week^−1^	108.8 ± 32.9	106.1 ± 36.6 **	91.7 ± 33.2	85.6 ± 20.8	**0.018**
VO2 max, mL/kg/min	62.6 ± 15.4	53.0 ± 6.9 *	55.7 ± 8.4	47.1 ± 8.6	**0.011**
VO2 max, L/min	4.44 ± 0.81	3.21 ± 0.67	4.29 ± 0.59	3.03 ± 0.49	0.295
Peak torque, ft-lbs	114.4 ± 26.2	65.5 ± 12.8	124.2 ± 24.5	73.6 ± 18.6	0.104

^1^ Data are the mean ± SD; n in parentheses; gender distribution did not differ by diet group (*p* = 0.460; Chi Square analysis). *p* for 2-way ANOVA analyses by diet (non-normal data transformed prior to analysis (visceral fat)). The single asterisk (*) indicates significant difference within gender by diet group (*p* < 0.05); the double asterisk (**) indicates a trend for difference within gender by diet group (0.05 < *p* < 0.10).

**Table 2:** Nutrient differences by diet group (vegetarian, VEG; omnivorous, OMN) ^1^.

	VEG (22)	OMN (35)	*p*	Reference Range ^2^
Total kilocalories (kcal)	2443 ± 535	2266 ± 612	0.072	-
Carbohydrate (CHO) (g)	328 ± 70	248 ± 101	**0.001**	-
CHO (% energy)	53 ± 6	48 ± 7	**0.010**	45%–65%
Fiber (g)	38 ± 13	24 ± 9	**<0.001**	38/25 g [M/F]
Protein (g)	78 ± 19	101 ± 35	**0.006**	-
Protein (% energy)	12 ± 2	17 ± 4	**<0.001**	10%–35%
Protein (g/kg body mass)	1.2 ± 0.3	1.4 ± 0.5	0.220	0.8 g/kg
Fat (g)	90 ± 26	83 ± 33	0.901	-
Fat (% energy)	32 ± 5	32 ± 6	0.952	20%–35%
Saturated fat (g)	22.8 ± 11.2	25.7 ± 10.1	0.207	-
Saturated fat (% energy)	8.3 ± 3.1	11.6 ± 6.3	**0.002**	<10%
Cholesterol (mg)	102.8 ± 119.5	301.2 ± 165.6	**<0.001**	-
Vitamin C (mg)	117.0 ± 64.0	83.0 ± 46.5	0.076	90/75 mg [M/F]
Vitamin D (IU)	115.4 ± 111.4	129.0 ± 115.5	0.201	600 IU
Vitamin B12 (mcg)	3.0 ± 3	4.8 ± 4.6	**0.006**	2.4 mcg
Selenium (mcg)	41.8 ± 36.0	62.6 ± 33.6	**0.002**	55 mcg
Sodium (mg)	2931.2 ± 783.1	2972.8 ± 887.5	0.794	<2300 mg
Iron (mg)	19.4 ± 7.8	15.4 ± 5.4	**0.017**	8/18 mg [M/F]
Zinc (mg)	8.5 ± 9.1	8.9 ± 4.9	0.149	11/8 mg [M/F]
Calcium (mg)	971.0 ± 401.6	878.1 ± 314.9	0.378	1000 mg
Phosphorus (mg)	782.0 ± 378.0	831.2 ± 336.4	0.507	700 mg
Omega-3 fatty acid (g)	1.6 ± 2.5	0.9 ± 0.7	0.326	-
Omega-3 fatty acid (% energy)	0.004 ± 0.005	0.004 ± 0.003	0.613	0.6%–1.2%
Omega-6 fatty acid (g)	7.7 ± 5.4	6.1 ± 4.4	0.145	-
Omega-6 fatty acid (% energy)	2.8 ± 1.6	2.4 ± 1.3	0.358	5%–10%

^1^ Data are the mean ± SD; sample size in parentheses. *p* for general linear model analyses (non-normal data transformed prior to analysis (all variables except carbohydrate variables and fat percentage) and 2 outliers (VEG group) removed prior to analysis for saturated fat); ^2^ Reference ranges are the Recommended Dietary Allowance or the Acceptable Macronutrient Distribution Range; note the American College of Sports Medicine recommends that athletes consume 1.2–2.0 g protein/kg body mass.
